# Implementing the human right to science in the context of health-related data processing

**DOI:** 10.1093/jlb/lsae004

**Published:** 2024-03-16

**Authors:** Fruzsina Molnár-Gábor

**Affiliations:** Legal Faculty, BioQuant Centre, Heidelberg University, Im Neuenheimer Feld 267, 69120 Heidelberg, Germany

**Keywords:** health-related data processing, human right to science, supranational implementation, GDPR

## Abstract

This paper contributes to the exploration of the potential *application of duties* related to the diligent anticipation of the (imminent) harms and (potential) benefits to humans that scientific innovation engenders to health-related contexts. In particular, it addresses the intersection between the human right to science and health-related data processing, which plays a key role in the production, translation and implementation of biomedical knowledge. The first part of the paper provides a brief recap of the interpretation of the right to science based on Art. 15 (1) (b) of the United Nations International Covenant on Economic, Social and Cultural Rights (hereafter ICESCR or Covenant) and the resulting obligations for States in the context of health and related data processing. The second part of the paper defines the relevance of the ICESCR for EU Member States and the European Union. In the third part, theses are put forward on how the human right to science and the obligations under Art. 15 (1) (b) ICESCR influence the interpretation and application of the General Data Protection Regulation as secondary EU law. By examining the justifications for using the right to science to interpret EU data protection law and by providing interpretation and application guidance on the main data protection principles in the area of health-related data processing, taking this right into account, the aim is to shape the EU data governance framework to meet the requirements of this human right. In doing so, the paper aims to close the gaps in the interpretation and application of the main rules of EU data protection law. Such standardization in the health-related context can contribute to a coherent interpretation and application of existing rules by referring to this emerging human right. Against this background, the paper identifies governance measures that the EU legislator could take to guide the processing of health-related data in line with the requirements of the right to science.

## I. INTERPRETATION OF THE RIGHT TO SCIENCE IN THE CONTEXT OF HEALTH-DATA PROCESSING: ART. 15 (1) (b) ICESCR: THE RIGHT TO ENJOY THE BENEFITS OF SCIENTIFIC PROGRESS AND ITS APPLICATION

### I.A. Background of Interpretation

Hidden at the end of the list of rights in Part III of the United Nations International Covenant on Economic, Social and Cultural Rights (hereafter ICESCR or Covenant), Art. 15 ICESCR received little of the attention it deserved until 2020, when the Committee on Economic, Social and Cultural Rights (CESCR), the treaty-monitoring body of the Covenant, published General Comment (GC) No. 25.^1^ The GC follows earlier analyses of the article by the UN Special Rapporteur,^2^ as well as contributions from academia.^3^ This culminating attention to the article has contributed significantly to a higher level of consensus on the interpretation of the precise meaning of the components of the right to enjoy the benefits of scientific progress and its application (hereafter the right to science) enshrined in Art. 15 (1) (b) ICESCR, including its relevance to specific areas of science such as health-related data sciences.

Before outlining the interpretation of the elements of the right to science, it is necessary to anticipate the significance of GC No. 25. Treaty monitoring bodies interpret the provisions of a treaty in accordance with Art. 31 and Art. 32 of the Vienna Convention on the Law of Treaties (VCLT) and give concrete meaning to the rights of the individual and the obligations of the State.^4^ A dynamic approach to human rights norms^5^ that recognizes and prescribes a regime of legal protection fed by a ‘living’ interpretation of treaties^6^ presupposes a degree of interpretative discretion for treaty bodies within the confines and object of the particular treaty. At the same time, the parties’ discretion with respect to the *domaine réservé* regularly prevents treaty bodies from adopting overly surprising interpretations of an international treaty.^7^ GC No. 25 demonstrates this ambiguous balancing act between dynamic treaty interpretation and State sovereignty, leaving the state of the art of scientific inquiry and of the translation of its findings to guide interpretation, as in the field of health.

### I.B. Scientific progress and its Application

According to the 1974 UNESCO’s Recommendations on the Status of Scientific Researchers,^8^ science describes the organized attempt of individuals and groups, and generally and intrinsically by humankind, to reflect on and conceptualize phenomena through systematic and method-driven objective inquiry, in order to discover and master causalities and sort knowledge subsystems.^9^ Respectively, science is about the methodological sound creation of knowledge with a general purpose, and is subject to legal regulation.^10^

Science is the ultimate goal of the exercise of the right to freedom of science. For this reason, it is of particular importance that Art. 15 (3) ICESCR refers to the freedom essential to the exercise of scientific activities^11^ and places the right in its social context by highlighting two of its aspects.

First, science is to be understood in a broad sense encompassing physical, experimental, natural sciences and social sciences, humanities, philosophy and traditional as well as indigenous knowledge.^12^ Without prescribing a particular concept of science or scientific theory, science in the sense of Art. 15 (1) (b) ICESCR is all research activities, including necessary preparatory and transformative measures for the pursuit of scientific activities.^13^ Along these lines, data science, as a technological science, can not only map reality but also shape it at a general level while contributing to research collaboration and communication of methods and results. Its applications have recently stood out in the health field through the particular implementation of its results in response to specific concerns and needs of society, such as the translation of basic science results to specific areas of health care and medicine. Data science applied to health also includes technology derived from scientific findings, such as medical applications or information and communication technology for data sharing.^14^ Particularly health data sharing in the field of genomic medicine have led to significant advances in understanding and treating disorders and diseases,^15^ and AI-powered health applications will continue to express the data-driven nature of health science in the future.

Second, only scientific research aimed at expanding knowledge and analyzing various findings with intellectual autonomy deserves legal protection – there is an ethical condition inherent in human rights protection that shall guide the setup, method, conduct and application of scientific endeavours, and the application of its results.^16^ This ethical condition also stipulates that science must work for the benefit of humanity, respect research participants, and implement their rights.^17^ Accordingly, the advancement of science is an endeavour whose substantive and procedural elements of realization are guided by ethical principles while being subject to a freedom that may be constrained by the human rights system.^18^

### I.C. To Enjoy the Benefits

According to GC No. 25, the right to science includes not only the right to receive the benefits of the applications of scientific progress, but also the right to participate in scientific progress.^19^ Participation structures occur in two forms: The participation of the individual as Everyman (and thus indirectly of the general public) in the results of scientific development, and the participation of the scientifically active person in his own (and others’) findings.^20^ Related to this, although the benefit is primarily defined in material terms, according to GC No. 25 it also refers to knowledge and information (immaterial benefits) and to the role of science in the education of citizens.^21^

The interconnectedness and interdependence of all human rights directly affects what kind of scientific work is purposeful for receiving benefits from scientific progress and for participating in scientific progress. First and foremost, respect for human dignity, non-discrimination and equal treatment, and a focus on the disadvantaged can be directly mandated.^22^ In addition, human rights particularly related to the right to science, such as the right to life, the rights of the child, and the right to health, come to the fore.^23^ The latter is less defined as a binding legal right, but rather framed as a ‘right to health care’ and a ‘right to health protection.’ According to GC No. 14, ‘the right to health embraces a wide range of socio-economic factors that promote conditions in which people can lead a healthy life’.^24^

The right to science and the right to health are linked in a special way in the context of data science. Scientific knowledge based on appropriate health data can contribute to more efficient, higher quality, safer, and more stratified diagnoses and therapies, thereby impacting the well-being and lives of individual patients and the patient community. This link is also evident in GC No. 14, e.g., through the reference to disease control, which emphasizes individual and collective efforts by States to, among other things, provide relevant technologies, use and improve epidemiological surveillance and data collection on a disaggregated basis; or through the reference to commitments related to promoting recognition of factors that promote positive health outcomes, e.g., research and provision of information.^25^

Overall, the premises of interconnectedness and interdependence of human rights lead the right to science and the right to health to be mutually reinforcing, and the obligations arising from these rights should be understood as promoting the human rights system as a whole. Such promotion includes respecting and prioritizing interpretations of obligations that benefit different perspectives on the protection of individuals. Furthermore, any interpretation of human rights and related obligations that would block other human rights or weaken related obligations must be set aside.^26^

### I.D. Elements of the Right

The main elements of the right from the perspective of health-data processing can be outlined as follows.^27^

With respect to availability, the mandate is characteristic that States Parties should use their own resources and coordinate the actions of others to ensure that scientific progress occurs and that its applications and benefits are disseminated and available. This requires, among other things, a strong research infrastructure with adequate resources, which should include data research infrastructures. In particular, research results and research data funded by the States should be available to the public.^28^

With regard to the accessibility of scientific progress and its applications to all people, without discrimination, three dimensions are highlighted.^29^ First, States Parties should ensure that all people have equal access to the applications of science, particularly where these are relevant to the enjoyment of other economic, social and cultural rights, which include the right to health. This could mean applying access to data-driven health applications and modalities for providing data for their use. It also means that there should be modalities for making data available for scientific research. Second, this obligation is expanded by the provision that information about the risks and benefits of science and technology should also be accessible without discrimination. Transparency about the risks of data processing is one of the most important perspectives of non-discriminatory data processing in the health context, from which informed consent and information requirements derive. Third, by stating that everyone should have the opportunity to participate in scientific progress, including without discrimination, respect should not be left behind for research participants and patients whose opportunities for participation, as well as limitations on their rights such as intrusions into their privacy, depend on more complex modalities like those of patients with rare diseases.

Quality refers to the most advanced, current, generally accepted, and verifiable science according to standards generally accepted by the scientific community, both in the process of scientific creation and in access to applications.^30^ For health data science, this means that FAIRness of research data,^31^ protected by the state of the art in technical and organizational measures, should be understood as a value and a condition. Quality specifications in data science are often implemented through certification measures and through the standardization of approval procedures, such as the evaluation of research projects from an ethical point of view.^32^ In addition, there is a need to promote the sharing of health data among public stakeholders by requiring States to rely on generally accepted scientific evidence and to regulate and certify, in dialogue with the scientific community, the dissemination of new scientific applications that are available to the public.^33^

Acceptability requires that benefits to research participants and other data subjects be maximized and potential harms minimized through appropriate protections and safeguards; autonomy and free and informed consent of participants should be ensured; privacy and confidentiality should be respected; vulnerable groups or individuals should be specially protected to avoid any discrimination; and cultural diversity and pluralism should be adequately addressed.^34^ This mandates risk assessment and proportionality in data processing and already points to multipolar trade-offs in health data science and translational medicine (see below).

The reference to freedom of science does not further clarify the status of Art. 15 (3) ICESCR, so that it is not directly possible to draw conclusions about any entitlement to research support services, let alone research funding.^35^ However, the right to freedom of science according to Art. 15 (3) ICESCR in conjunction with Art. 9 (2) of the International Covenant on Civil and Political Rights (ICCPR) establishes, as far as possible, science-adequate promotion of research by public institutions. Considering that basic and translational health data research increasingly relies on high validity (based on comprehensive datasets) as well as on cross-border collaboration, to name just two crucial cornerstones, meeting the needs of science must also include measures to promote data sharing in scientific collaboration.

## II. APPLICATION OF DUTIES

### II.A. Duties

#### II.A.1. General obligations

Part II of the ICESCR identifies general obligations of the State that apply to all rights defined in the Covenant, while Part III of the ICESCR refers to specific obligations with respect to particular rights.^36^

With regard to general obligations, the central norm is Article 2 (1) ICESCR, according to which each State Party undertakes to work, individually and through international assistance and cooperation, in particular economic and technical assistance and cooperation, within its means (to the maximum of its available resources), towards the progressive realization of the rights recognized in the Covenant by all appropriate means, including in particular the enactment of legislation.

The cornerstones of this provision are progressive realization, international assistance, and cooperation, which are instructive for both the active promotion of rights and compliance with the negative obligations that flow from them.^37^ While progressivity relieves Parties of the immediate burden of realizing obligations, by reference to the active, positive obligation, and in combination with ‘all appropriate means’ where immediate effect can be achieved through action, delays and restrictions by Parties could constitute interference with Covenant rights.^38^ In addition, ‘all appropriate means’ refers to legislative measures, but beyond that, it also refers to other measures such as the formulation and implementation of national policies.^39^ It also mandates the provision of the ‘maximum available resources’, giving Parties discretion depending on their economic and financial capabilities, while requiring them to ‘strive to ensure the widest possible enjoyment of the relevant rights under the prevailing circumstances’.^40^

Direct and indirect links between the right to science and (the right to) health and the right to science and data sharing can be demonstrated in GC No. 25 in the context of general obligations.

With reference to para 70, which requires States Parties to provide and make available to all without discrimination the best available applications of scientific progress necessary to enjoy the highest attainable standard of health, and in conjunction with para 23, which requires that while the full realization of the right to science may be achieved progressively, steps in that direction must be taken immediately or within a reasonably short period of time, the creation of data-sharing infrastructures could be seen as one possible means of creating the conditions for fulfilling obligations.

Although national budgets may be limited, international cooperation may be a good way to expand local capabilities that depend on knowledge, infrastructure, and the provision of the framework needed for rights enforcement in the context of health-related data processing. This could even lead to an obligation in the context of health-related data sciences, to seek technical assistance from other, more developed States or the international community when States are unable to meet their obligations due to limited resources.

#### II.A.2. Specific obligations

The specific obligations can be divided according to the triad of measures of respect, protection, and fulfilment.^41^ The first prohibits any violation by State authorities of the rights enshrined in the Covenant for any citizen under the jurisdiction of the State, and is often summarized by describing the rights as negative rights in the sense of freedom from State interference.^42^ The duty to protect requires States to take measures to protect their citizens from violations by (private) third parties.^43^ The duty to fulfil requires the State to play an active role, whether in the form of legislation, administrative, budgetary, judicial, or other measures.^44^

In particular, in the context of data processing, among the respect obligations, the refraining from imposing or removing obstacles to international cooperation among scientists should be highlighted, unless such restrictions can be justified in accordance with Art. 4 of the Covenant.^45^ Among the obligations to protect, the requirement for States Parties to take measures to prevent persons or entities from interfering with the right to participate and enjoy the benefits by, for example, preventing access to knowledge, protecting people from participating in research or testing that violates applicable ethical standards for responsible research, and guaranteeing their free, prior, and informed consent has to be emphasized.^46^ Among the obligations to be fulfilled, the requirement that States adopt legislative, administrative, budgetary, and other measures aimed at ensuring the full enjoyment of the right to science includes providing frameworks for the participation in international cooperation programs and ensuring adequate funding.^47^ From the perspective of data sharing, the importance of the duty of States to disseminate science and promote citizen participation should not be underestimated. Fundamental scientific methods and results, which have become an essential element for the exercise of citizenship and other rights, oblige States to make every effort to ensure equitable and open access to scientific literature, data, and content, including by removing barriers to the publication, sharing, and archiving of scientific results.^48^

#### II.A.3. Core obligations

Among the core obligations listed in the GC, the following seem particularly relevant to data sciences in the health sector: (i) The elimination of laws, policies and practices that unjustifiably limit access by individuals or particular groups to information related to science, scientific knowledge and its applications; (ii) The removal of limitations to the freedom of scientific research that are incompatible with Art. 4 ICESCR; (iii) The adoption and implementation of a participatory national strategy or action plan for the realization of the right to science that includes a strategy for the development of science; (iv) Ensuring access to those applications of scientific progress that are critical to the enjoyment of the right to health and other economic, social and cultural rights; (v) Ensuring that in the allocation of public resources, priority is given to research in areas where there is the greatest need for scientific progress including in health; (vi) Adoption of mechanisms aimed at aligning government policies and programmes with the best available, generally accepted scientific evidence; (vii) Ensuring that health professionals are properly trained in using and applying modern technologies and medicines resulting from scientific progress; (viii) Fostering the development of international contacts and cooperation in the scientific field, without imposing restrictions on the movements of knowledge beyond those that are justifiable in accordance with Art. 4 ICESCR.^49^

#### II.A.4. Precaution generally and with regard to emerging technologies and international cooperation

For data sharing and processing, the interpretation of the precautionary principle is of particular importance. This principle requires that certain risks of data processing must be accepted in order to avoid unacceptable harms, and that proportionality must be maintained when balancing competing interests.^50^ With respect to proportionality, the list of unacceptable harms is of particular interest and includes harms to humans that (i) pose a threat to human life or health, (ii) are severe and effectively irreversible, (iii) are unfair to present or future generations, or (iv) are imposed without due regard to the human rights of those affected.^51^ The GC promotes technology and human rights impact assessments as tools for early identification of potential risks, as well as the use of scientific applications. In addition, it can be mentioned that technical measures of data processing and the organizational measures for handling them can contribute to maintaining the proportionality of data processing among many data protection regulations, which can essentially be determined on a risk basis. Ultimately, the targeted risk orientation of data protection regulations can contribute to a balanced implementation of the precautionary principle. With regard to emerging technologies, States Parties must adopt policies and measures that expand the benefits of these new technologies while reducing their risks.^52^ For the Committee, two elements remain very important: first, strengthening international cooperation in this area to create the conditions for global regulations and avoid fragmented national responses that would create governance gaps detrimental to the rights of the Covenant and perpetuate technological and economic inequalities.^53^ Second, decisions about the development and use of these technologies should be made within a human rights framework and from a holistic and inclusive perspective.^54^ All of the overarching human rights principles of transparency, non-discrimination, accountability, and respect for human dignity are critical in this area. These principles correspond to the key data protection law principles established in a multi-level regulatory environment.^55^

With regard to international cooperation, States must take action through legislation and policy to create a global environment that fosters the advancement of science and the benefits of its applications.^56^ Increased international cooperation is cited as an example of how States and international organizations could be better prepared for future pandemics by sharing scientific information on potential pathogens, improving early warning mechanisms based on timely and transparent information from States on emerging epidemics to provide the best science for intervention, and sharing the best science and its applications, particularly in the medical field, to mitigate the impact of disease and accelerate the discovery of effective treatments and vaccines once a pandemic has developed.^57^ These provisions also prescribe frameworks for data sharing and provision in health research and translational contexts. In addition, the extraterritorial obligations of States are emphasized.^58^ Particularly forceful is the provision reminding that States Parties involved in decision-making as members of international organizations, which from an international law perspective includes the EU, cannot ignore their human rights obligations.

### II.B. Roots to Implement Duties

#### II.B.1. National implementation

In line with Art. 26 VCLT, GC No. 9 on the domestic application of the Covenant emphasizes that ‘the central obligation in relation to the Covenant is for States Parties to give effect to the rights recognized therein’.^59^ With respect to the means of implementation, the Covenant allows States Parties to decide on the form and procedure by which they will meet central obligations, depending on how the domestic legal system deals with public international law and its treaties.^60^ In any case, in compliance with Art. 27 VCLT, existing domestic law cannot be used as a justification for a State’s failure to comply with its obligations under the Covenant.

#### II.B.2. Supranational implementation

Art. 2 TEU states that the EU is founded on the values of respect for human dignity, freedom, democracy, equality, the rule of law and respect for human rights. Art. 3 (5) TEU emphasizes that in its relations with the wider world, the EU shall uphold and promote its values and interests and contribute to the protection of its citizens and that it shall contribute, inter alia, to the protection of human rights, as well as to the strict observance and the development of international law.

In binding the EU to treaties under international law, a distinction must be made between treaties to which the EU itself is a party and those to which its Member States alone are parties. The EU is bound by international law only in the first case and is not (directly) bound by international treaties of the Member States, even if all Member States are party to them. In these cases, a binding obligation under international law can only be established by a formal accession of the EU to the international treaty or by an informal legal succession.^61^

However, even without being bound by international law, international treaties can take on significance in the legal order of the EU. Contractual obligations of the Member States serve the Court of Justice of the European Union (CJEU), on the one hand, as a source of legal knowledge for the development of general legal principles of EU law.^62^ On the other hand, under certain conditions, there is an even more far-reaching obligation for treaties from the time before the establishment or accession to the EU of a Member State that is a party to the respective treaty under international law. If the matters regulated in such a treaty have been transferred to the competence of the EU by the conclusion of the EU Treaties or their further development, the CJEU recognizes as an expression of the will of the Member States to bind the EU to the relevant contractual obligations of the Member States.^63^

In addition, there exists the special provision of Art. 351 of the Treaty on the Functioning of the European Union (TFEU) for old treaties of the Member States. According to Art. 351 (1) TFEU, rights and obligations arising from treaties concluded by Member States before their establishment or accession to the EU are not affected by the EU Treaties.^64^ On the other hand, Art. 351 (2) TFEU imposes obligations on the Member States concerned to remedy any incompatibilities between the EU Treaties and the obligations previously entered into (eg, through adaptation or withdrawal).^65^

The law of the EU is that of the international legal order, the main sources of which is International Treaty Law, customary international law and general principles of law. The rules of customary international law are part of the law of the EU.^66^ In case of conflict of laws, the primary law of the EU prevails over the general international law as a more specific rule. On the contrary, general international law takes precedence over secondary EU law. In relation to the right to science, this means that its codification by Art. 15 (1) (b) ICESCR in international treaty law, as well as its codification in Art. 27 of the Universal Declaration of Human Rights in customary international law,^67^ are instructive for the interpretation of secondary EU law such as the EU General Data Protection Regulation (GDPR). Additionally, the right to science gains influence when defining the general principles of EU law by the CJEU, inter alia, which include fundamental human rights determining the lawfulness of legislative and administrative measures within the EU.

## III. IS THE INTERPRETATION OF THE GDPR IN COMPLIANCE WITH THE RIGHT TO SCIENCE?

Based on the bindingness of the right to science for the EU with regard to its secondary law, in the last step, selected developments in the interpretation of the GDPR in the context of health-related scientific data processing will be challenged. The following theses have been subject to critical discussion in the workshop.

### III.A. Data Processing as a Prohibition Subject to Authorization

Based on the application of the right to science of Art. 15 (1) (b) ICESCR, which is subject to the principle of proportionality under GC No. 25, it is to be included in the assessment of the design and limitations of the core fundamental right in the context of health-related data sharing in EU secondary legislation, which is the fundamental right to data protection under Art. 8 (1) Charter of the Fundamental Rights of the EU (CFREU). The legal position in the trade-off between privacy and data processing interests becomes multipolar from the perspective of data subjects with regard to enjoying the benefits of scientific progress. Particularly in the field of translational medicine, ie, the translation of scientific research results to the clinical area, a pronounced interest in participating in health research compared to other contexts of scientific research is substantiated due to the potential relevance of research results on a personalized and stratified level. This relevance is to be seen under the umbrella of the interconnectedness and interdependence of human rights and accordingly with respect to the interdependence of the right to health and the right to science.

Within the weighing that is multipolar from the perspective of data subjects with competing legal interests in their very own legal positions, eg, privacy vs. right to science, the secondary design of data protection as defined by the provisions of the GDPR is to be understood as the manifestation of the operationalization of the trade-offs of the complex weighing that is defined on an abstract-general level through the regulation and will be refined and further detailed in the course of law application to provide single-case justice.

Data processing can be described as the set of actions that need to be exercised to achieve a certain scientific research aim compliant with human and fundamental rights as well as legislators’ legitimate balancing of competing fundamental and human rights positions. Accordingly, the assumption that data processing can generally be prohibited, cannot be upheld if such an assumption excludes human and fundamental rights positions that are attached to the promotion and exercise of data processing, including the data subjects’ right to science.

Refusing the interpretation of data processing as subject to a general ban does not go against EU primary law which needs to be respected when thinking about the binding effect of treaties of EU Member States to which the EU itself is not a party to.

The ‘prohibition principle’ is linked to the principle of lawfulness, Art. 5 (1) (a) GDPR. Thus, it is argued that the principle of ‘lawfulness’ means the principle of prohibition with reservation of permission.^68^ The fact that Art. 6 (1) subpara. 1 GDPR makes lawful data processing dependent on the fulfilment of one of the conditions mentioned there, is considered by many to be a ‘prohibition with reservation of permission’.^69^

For data protection, insofar as the CFREU is applicable, Art. 7 and 8 CFREU are relevant. Art. 8 CFR specifically protects the data subject’s decision-making power over his or her personal data.^70^ According to its paragraph 1, every person has the right to the protection of personal data concerning him or her. Art. 8 (2) subpara. 2 CFREU requires that data processing must be for a specific purpose, and grants a right to information and rectification. Art. 8 (3) CFREU stipulates that an independent body must monitor data protection.

The fundamental rights in Art. 7 and 8 (1) CFREU contain rights of defense in a classic sense against public responsible parties. However, since fundamental rights holders are part of a society that relies on processing of personal data, they must, in the overriding public interest, accept legitimate interference with their fundamental rights.^71^ To the extent that non-public controllers interfere with fundamental rights, Art. 7 and Art. 8 CFREU oblige legislators and public authorities to protect these fundamental rights against interference by private parties. States have the positive obligation to protect the fundamental rights of citizens against the exercise of fundamental rights by other citizens as well.^72^

Art. 8 (2) (1), subpara. 2 CFREU gives concrete form to the general legal reservation of Art. 52 (1) CFREU for the fundamental right to data protection, without distinguishing between the public and non-public spheres.^73^ Furthermore, it requires that the law may only permit data processing if it is in good faith, if it provides for purpose specification and purpose limitation, grants every person a right of information, right of access and rectification, and that data processing be subject to the control of an independent body. This is how the principle of lawfulness in Art. 5 (1) (a) GDPR is to be understood.^74^ The regulation on the permissibility of data processing in Art. 6 (1) GDPR follows the principle that fundamental rights in particular may only be interfered with if the legislator has permitted them by law.^75^ Furthermore, Art. 9 (1) GDPR also does not contain a constitutive prohibition, but only clarifies that the abstract permissive elements in Art. 6 (1) GDPR do not apply to the special categories of personal data. Rather, for the processing of special categories of personal data referred to in Art. 9 (1) GDPR a special permission listed in Art. 9 (2) GDPR must additionally be drawn on.^76^

Thus, the legislator does not prohibit an encroachment on fundamental rights, but rather allows encroachments on fundamental rights through data protection law on the basis of their social necessity or desirability. The permissibility is based on balancing the fundamental right to data protection with conflicting interests. Insofar as the fundamental rights of data controllers or the public interests pursued by data processing are predominant, data processing can take place.^77^ It is thus released from any general ban.

According to Art. 52 (1) CFREU, limitations on the right to data protection must be provided for by law and respect the essence of the rights and freedoms concerned. In compliance with the principle of proportionality, restrictions may only be imposed if they serve objectives which are recognized by the EU as being in the public interest, or the protection of the rights and freedoms of others and are necessary for this purpose.

### III.B. Rethinking the Role of Risk Assessment of Data Processing

According to the principle of proportionality, which should apply to restrictions on the right to science, this test should always be at the forefront when it comes to restricting this right, such as through provisions under data protection law. This means that a risk assessment must be carried out throughout a data processing initiative. This includes assessing the scope of application of provisions restricting the right to science, such as anonymity triggering a limited usability of data for scientific purposes and translational medicine, assessing possible interference with the rights and freedoms of data subjects and deciding on the lawfulness of international data transfers.

#### III.B.1. Identifiability and its effects on the protection of data subjects

Overall, the critical factor is how likely it is that the data will be established as personally identifiable. Only those means that can ‘reasonably’ be used to establish the personal link are to be considered.^78^ The classification of data as ‘anonymous’ or as ‘non-personal’ thus depends on the intensity of the effort required to establish a reference to a person.^79^ The assessment of the intensity in turn depends on the reasonableness threshold: The effort required to establish a reference to a person must not be disproportionate. The use of technical measures that enable the establishment of the personal reference, such as the ‘singling out’ referred to in recital 26 sentence 3 GDPR, must also be reasonably applicable. The mere facts that measures exist that enable the establishment of the personal reference does not mean that they can be used with a proportionate effort.^80^

The assessment of likelihood of (re-)identification can be done using risk prediction, taking into account both the factors inherent in the potential data processor itself, such as its knowledge, and the means of attribution that can be used by data processors.^81^ In practice, this is usually done by assuming a maximum level of risk and assessing the likelihood of occurrence using objective factors that can be applied on the basis of a general discretion (reasonability).

The risk to the rights and freedoms of the data subject determines the manner of data processing. In this context, the risk of (re-)establishing a reference to a person and the risk of processing for the rights and freedoms of the data subjects are to be seen as interdependent predictions that can be combined into a unified impact assessment of data processing.^82^

Reducing the richness of data through anonymization techniques diminishes the scientific and public health value of the data and limits the potential for answering questions in the context of research and care, applied research and diagnostic methods, and the relevance of research findings.^83^ Therefore, it also plays a role in determining appropriate technical and organizational measures for data processing that serve the practical implementation of proportionality. The different legal interpretations of anonymity affect the evaluation of the technical implementation of de-identification and may thereby affect the usefulness of datasets for probands and patients with regard to health-related findings, translational, stratified medical measures and ultimately healthcare and treatment, and thus the benefits from science for those effected.

#### III.B.2. International transfers

The interpretation of adequacy must be guided by the right to science, which can, in first place be provided by implementing the proportionality principle to the evaluation of international data transfers, prescribing a risk assessment of envisaged transfers. With regard to Chapter V GDPR which contains the rules for international data transfers, this should be assessed in light of Art. 4 ICESCR. This provision allows limiting the rights of the Covenant, under the condition that the limitations are determined by law only in so far as this may be compatible with the nature of the rights and solely for the purpose of promoting the general welfare in a democratic society. Thus, limitations are subject to the principle of proportionality, which means that the protection of rights must not be levelled by invoking the Covenant. The core of the rights of the Covenant must be preserved at all times.

For international data transfers, Chapter V of the GDPR provides for a two-step assessment. The first stage examines whether the data are processed lawfully, and the second stage examines whether the chosen data transfer mechanisms comply with the provisions of Chapter V of the GDPR.^84^ The provisions of Art. 44 et seq. GDPR provide that the data transfer provisions of Chapter V of the GDPR are to be applied in a way that does not undermine the level of protection provided for in the Regulation. This is ensured if the level of data protection in the third country is ‘adequate’. The level of protection in the third country is adequate if it is actually equivalent in substance to the level guaranteed in the EU due to its national legislation and international obligations.^85^ Accordingly, an identical level of protection is not required, but the level of protection must be functionally close to that provided by EU law.^86^ The assessment of adequacy is thus based on the level of data protection of the third country compared to that of the Charter of Fundamental Rights and its secondary law concretized by the GDPR.

The design of the transfer is thus also based on appropriate safeguards for the rights of the data subjects, which are to be measured against the principle of proportionality.^87^ This is either established by a decision of the European Commission for a third country or a sector in a third country or ensured by the use of predefined transfer mechanisms.^88^ In the latter case, if the assessment of the level of data protection in the third country reveals that the legislation and/or practices there compromise the effectiveness of the transfer mechanisms chosen by the data exporter in the context of Art. 46 GDPR and thus call into question the adequacy of the data processing in the third country, additional measures must be taken.^89^ The additional measures serve the purpose of compensating for the lack of adequacy of the level of data protection in the third country.^90^ Through this compensatory effect, they can ensure the proportionality of the data processing by minimizing the risks to the rights and freedoms of the data subjects.

The main objective of the GDPR is to provide a framework in which the controller balances the risks of processing personal data and the protection of the interests of the data subject with the interests of the processor. The GDPR’s mechanisms for international data transfers are intended to ensure that the requirements applicable under EU data protection law remain effective after data is transferred outside the EU and the European Economic Area, including the proportionate balancing of benefits and harms of processing.

Restrictions on international data transfers in the health context, and thus indirectly on data subjects’ right to research, are not unnecessary with respect to surveillance activities by third country authorities if those activities violate the essence of the right to data protection. As stated by the CESCR in its concluding observations on Canada in 2006, ‘restrictions should be eliminated where they are not strictly necessary for the promotion of the general welfare in a democratic society, for the protection of the interests of national security or public safety, public order, public health, or the protection of the rights and freedoms of others, and where no other alternative can be found’.^91^ Moreover, according to GC No. 21 on the right to take part in cultural life, to which the right to science is closely related,^92^ ‘[s]uch limitations must […] be strictly necessary for the promotion of general welfare in a democratic society […]. Any limitations must therefore be proportionate, meaning that the least restrictive measures must be taken when several types of limitations may be imposed.’^93^

In considering that the restriction of this right must also not be disproportionate and must respect the nature of the right to science, a reasonability standard and objectivity is applied. Beyond assessing whether the aim and effects of the measures or omissions are legitimate, compatible with the nature of Covenant rights, and solely for the purpose of promoting the general welfare in a democratic society, there must be a clear and reasonable relationship of proportionality between the objective pursued and the measures or omissions and their effects. Failure to eliminate differential treatment because of the lack of available resources is not an objective and reasonable justification unless every effort has been made to use all resources available to the State Party to address and eliminate the discrimination as a matter of priority.^94^ As the CESCR noted in GC No. 25, the prohibition of discrimination applies immediately and is not subject to progressive implementation or dependent on available resources.^95^

The obligation of the EU organs to promote international data transfers as an essential tool for data sharing for the benefit of data subjects in the health context, which derives from their human right to science, sheds light on the lack of EU action to promote data sharing. For example, this could be done through legal instruments for data sharing with international organizations such as the WHO. The absence of such instruments sheds light on the obligation to create the possibility of processing personal data for health-related scientific endeavours in third countries where an adequate level of data protection is not given.

## IV. CONCLUSIONS: THE WAY FORWARD

### IV.A. Defining Obligations of the EU to Assist Member States in Fulfilling their Obligations Arising from the Right to Science

As for the obligations defined by the CESCR and derived in the above analysis for the health-data context, they can be supported by the EU. The CJEU derives from Art. 351 (1) TFEU the tacit obligation of the EU vis-à-vis the Member States not to obstruct the fulfilment of the obligations arising for the Member States from earlier agreements (principle of loyalty).^96^ In this sense, the EU institutions are required, as far as legally possible, to avoid a situation of conflict with the old treaties by interpreting EU law in a manner that is friendly to international law. This does not imply a restriction of the EU legislative power, but it does impede the enforcement of primary and secondary law by the EU institutions until a conflict has been resolved. The principle of loyalty, combined with the application of Member States’ international treaties to secondary EU law, can be condensed into the active implementation of measures for the EU to promote health data sharing in the implementation and application of secondary EU law.

### IV.B. Options for Governance Measures

Governance measures can promote the implementation of technical and organizational tools that enable privacy-compliant processing and sharing of data, with the favourable circumstances for their application being created by EU public actors. Federated technologies enable the joint analysis of datasets held in a decentralized manner.^97^ For example, different healthcare providers can jointly perform statistical analysis and develop machine learning models without sharing the underlying datasets. Only aggregated results or model updates are transmitted. In this way, each healthcare provider can set its own specifications for processing its data and maintain control over access.^98^ Secure data spaces are another technical and organizational measure. By helping to define the context of data processing, secure data spaces can help to minimize the risks of establishing a link to a person and thus compromising the rights and freedoms of data subjects.^99^ This will allow the right to science to be fully promoted. Their technical and legal design also contributes to the realization of the proportionality principle in the further course of data processing. The underlying proportionality balancing between the conflicting interests of data controllers, such as scientific researchers, and data subjects is particularly influenced in the health sector, compared to other processing contexts, by the fact that processing results are often only meaningful in relation to individuals, and data subjects may have a strong interest in the processing, as well as in having the results tied back to their person to benefit from health-related and translatable research findings.^100^ Secure data spaces could thus fulfil the function of ‘balancing rooms’ to implement the proportionality principle related to the right to science.^101^

Another tool for fostering the implementation of obligations related to the right to science is to promote standardization of trade-offs related to data processing by stakeholders involved in scientific research and translational efforts in health, for example, through codes of conduct. Stakeholders involved in data processing, whether public or private, can better evaluate trade-offs based on knowledge of the actual potential benefits of a particular piece of scientific research if a governance framework has been established that allows them to make the necessary trade-offs between multipolar legal positions, including those that are also inherently present in the position of data subjects.

Last but not least, establishing a governance framework for the process of meeting certain conditions in balancing the right to science with competing legal positions is a measure that respects case-by-case decision-making and the justice that must be ensured thereby. Such measures may include the promotion of ethical evaluation of research projects and coordination of the decision-making processes of ethics committees, as envisaged by the GA4GH,^102^ as well as the harmonization of approval by data protection supervisory authorities, as envisaged by the consistency procedures defined in the GDPR.^103^

Part IV of the Covenant contains provisions with regard to international monitoring and co-operation as well as the involvement of UN specialized agencies, and establishes the ECOSOC as the co-ordination authority supervising activities relating to implementation. States submit reports^104^ and ECOSOC encourages all relevant specialized agencies of the UN to play an active role in promoting the rights of the Covenant,^105^ including through the submission of reports and comments. In addition, Art. 23 ICESCR reaffirms the concept of international co-operation by, inter alia, specifying the international measures to be taken by States Parties to realize the rights recognized in the Covenant. Further fostering adherence to the provisions of human rights law in supranational organizations like the EU that are subject to public international law would be a convincing start.

### IV.C. Summary

The loyalty principle and Member States’ international treaties (as applied to secondary EU law) can be realized through measures for the EU to promote health data sharing in the creation and implementation of secondary EU law. In addition, governance measures such as secure data spaces and standardization of trade-offs in relation to data processing by actors involved in scientific research and translational efforts in health, eg, through codes of conduct, are technical and organizational tools that enable data sharing in line with data protection and promote the lawful implementation of data sharing obligations and thus also the right to science. Stakeholders involved in data processing, often from the private sector, can better make trade-offs based on knowledge of the actual potential benefits of a given scientific research if a governance framework has been established that allows them to assess the interplay and weighing between multipolar legal positions. These instruments could thus fulfil the function of, or help to create, a balancing space and promote the implementation of the precautionary principle in the context of the right to science. Consequently, mitigating risks to data protection today should be done in such a way that the mitigation represents also the precaution for tomorrow in terms of securing the benefits from scientific progress.

## Footnotes

1 Committee on Economic, Social and Cultural Rights (CESCR), General comment No. 25 (2020) on science and economic, social and cultural rights (article 15 (1) (b), (2), (3) and (4) of the International Covenant on Economic, Social and Cultural Rights), 30 April 2020, E/C.12/GC/25, online: https://digitallibrary.un.org/record/3899847.

2 Human Rights Council, *Report of the Special Rapporteur in the field of cultural rights*, Farida Shaheed, The right to enjoy the benefits of scientific progress and its applications, 14 May 2012, A/HRC/20/26, online: https://documents-dds-ny.un.org/doc/UNDOC/GEN/G12/134/91/PDF/G1213491.pdf?OpenElement.

3 Instead of many, cf. Audrey R. Chapman, *Towards an Understanding of the Right to Enjoy the Benefits of Scientific Progress and Its Applications*, 8 Journal of Human Rights 1 (2009); Samantha Besson, *Science Without Borders and the Boundaries of Human Rights: Who Owes the Human Right to Science?*, 4 Eur J Human Rts 462 (2015); Sebastian Porsdam Mann, Helle Porsdam (eds.), The Right to Science: Then and Now (Cambridge University Press 2021); as well as many contributions by the participants of the workshop.

4 United Nations, Vienna Convention on the Law of Treaties, United Nations, Treaty Series, vol. 1155, p. 331, 23 May 1969, https://www.refworld.org/docid/3ae6b3a10.html [accessed 14 February 2024]. Kerstin Mechlem, *Treaty Bodies and the Interpretation of Human Rights*, 42 Vanderbilt Journal of Transnational Law 905, 919 (2009).

5
Malcolm D. Evans (ed.), International Law 690 (4^th^ ed. Oxford University Press 2014).

6 Daniel Moeckli, Nigel D. White, *Treaties as ‘Living Instruments’*, in Conceptual and Contextual Perspectives on the Modern Law of Treaties 136-171 (Michael J. Bowman, Dino Kritsiotis eds., Cambridge University Press 2018).

7 Eibe Riedel, *Sleeping beauty or let sleeping dogs lie? The right of everyone to enjoy the benefits of scientific progress and its applications (REBSPA)*, in Coexistence, Cooperation and Solidarity. Liber Amicorum Rüdiger Wolfrum, Vol. 1, 503-519, 516 et seq. (Holger P. Hestermeyer, Doris König, Nele Matz-Lück et al. eds., Martinus Nijhoff Publishers 2012). Concerning the differences between GC No. 25 and the Travaux Préparatoires, cf. Tara Smith, *Scientific Purpose and Human Rights: Evaluating General Comment No 25 in Light of Major Discussions in the Travaux Préparatoires of the Universal Declaration of Human Rights and International Covenant on Economic, Social, and Cultural Rights*, 38 Nordic Journal of Human Rights 221 (2020).

8 UNESCO, Recommendation on the Status of Scientific Researchers, UNESCO Gen. Conf. Res. 18 C/Res.40, 18th Sess. (1974).

9
*Id*. at I.1. (a)(i).

10
Matthias Ruffert, Sebastian Steinecke, The Global Administrative Law of Science 10 (Springer 2011).

11 With regard to the discussion of whether the formulation of Art. 15 (3) ICESCR as a State obligation can be read as an indication of the granting of an individual right, cf. *Id*., p. 32 et seq. Cf. also GC No. 25, 13 on the dimensions of this freedom, including “the protection of researchers from undue influence on their independent judgment; the possibility for researchers to set up autonomous research institutions and to define the aims and objectives of the research and the methods to be adopted; the freedom of researchers to freely and openly question the ethical value of certain projects and the right to withdraw from those projects if their conscience so dictates; the freedom of researchers to cooperate with other researchers, both nationally and internationally; and the sharing of scientific data and analysis with policymakers, and with the public wherever possible”.

12 GC No. 25, 5.

13
Matthias Ruffert and Sebastian Steinecke, The Global Administrative Law of Science 10 (Springer 2011).

14 GC No. 25, 7.

15 Ewan Birney, Jessica Vamathevan, Peter Goodhand, *Genomics in healthcare: GA4GH looks to 2022*, 203, 554.

16 UNESCO, Recommendation on Science and Scientific Researchers. Adopted on 13 November 2017, SHS/BIO/PI/2017/3, para. 5 (c), 12.

17
*Id*., Preamble (a).

18
*Id*., para. 16 (a) (v). Cf. Roberto Andorno, *The Right to Science and the Evolution of Scientific Integrity*, in The Right to Science: Then and Now 91-107, 102 (Sebastian Porsdam Mann, Helle Porsdam eds., Cambridge University Press 2021).

19 GC No. 25, 11 in conjunction with 9.

20 GC No. 25, 10.

21 GC No. 25, 8.

22 Cf. the preamble of GC No. 25, emphasizing every individual’s obligation towards the community in its quest for the full realization of the rights enshrined in the Covenant.

23 Accordingly, GC No. 14, 3 states that the right to health is closely related to and dependent upon the realization of other human rights, as contained in the International Bill of Rights, including, inter alia, privacy, access to information, and the freedoms of association, assembly and movement. These and other rights and freedoms address integral components of the right to health.

24 GC No. 14, 4.

25 GC No. 14, 37.

26 Audrey R. Chapman, *Towards an Understanding of the Right to Enjoy the Benefits of Scientific Progress and Its Applications*, 8 Journal of Human Rights 1 (2009); F. Valente, *The Right to Enjoy the Benefits of Scientific Progress and its Applications and the Right to Food*, in UNESCO Experts’ Meeting 55 (Amsterdam, 7–8 June 2007).

27 GC No. 25, 16 et seq. Critical evaluation: Yvonne Donders, *The right to enjoy the benefits of scientific progress: in search of State obligations in relation to health*, 14 Med Health Care Philos 371 (2011).

28 GC No. 25, 16.

29 GC No. 25, 17.

30 GC No. 25, 18.

31 Research data is FAIR, if it is Finable, Accessible, Interoperable and Re-usable. FORCE11 Consortium Guiding Principles for Findable, Accessible, Interoperable and Re-usable Data Publishing Version b1.0.https://www.force11.org/fairprinciples. Mark D. Wilkinson, Michel Dumont, IJsbrand J Aalbersberg, Gabrielle Appleton, Myles Axton M, Arie Baak et al., *The Fair Guiding Principles for scientific data management and stewardship*, 3 Sci. Data. 160018 (2016).

32 GC No. 25, 18.

33 GC No. 25, 18.

34 GC No. 25, 19.

35 GC No. 25, 20.

36 With regard to the relation between general and specific obligations, cf. Eibe Riedel, *International Covenant on Economic, Social and Cultural Rights (1966)*, in Max Planck Encyclopaedia of Public International Law recital 6 (Rüdiger Wolfrum, Frauke Lachenmann, Rainer Grote eds., April 2011). Cf. also Ben Saul, David Kinley, Jacqueline Mowbray, Covenant on Economic, Social and Cultural Rights. Commentary, Cases and Materials, 1214 et seq. (Oxford University Press 2014).

37 Yvonne Donders, *The right to enjoy the benefits of scientific progress: in search of State obligations in relation to health*, 14 Med Health Care Philos 371, 374 et seq. (2011).

38 GC No. 25, 23.

39 GC No. 25, 23.

40 GC No. 3, 11.

41 GC No. 25, 41, cf. also Eibe Riedel, *International Covenant on Economic, Social and Cultural Rights (1966)*, in Max Planck Encyclopaedia of Public International Law recital 14 et seq. (Rüdiger Wolfrum, Frauke Lachenmann, Rainer Grote eds., April 2011).

42 GC No. 25, 42.

43 GC No. 25, 43.

44 GC No. 25, 45.

45 GC No. 25, 42.

46 GC No. 25, 43.

47 GC No. 25, 45.

48 GC No. 25, 43, 49.

49 GC No. 25, 51 et seq.

50 GC No. 25, 53.

51 GC No. 25, 56.

52 GC No. 25, 72 et seq.

53 GC No. 25, 74.

54 GC No. 25, 75.

55
Lee A. Bygrave, Data Privacy Law. An International Perspective 145 et seq. (Oxford University Press 2014).

56 GC No. 25, 77.

57 GC No. 25, 82.

58 GC No. 25, 83.

59 GC No. 25, 9, 1.

60 Eibe Riedel, *International Covenant on Economic, Social and Cultural Rights (1966)*, in Max Planck Encyclopaedia of Public International Law recital 14 et seq. (Rüdiger Wolfrum, Frauke Lachenmann, Rainer Grote eds., April 2011).

61 See, for example, European Union, Treaty on European Union (Consolidated Version), Treaty of Maastricht, Official Journal of the European Communities C 325/5; 24 December 2002, 7 February 1992, https://www.refworld.org/legal/agreements/eu/1992/en/16882 [accessed 13 February 2024], Article 6(2).

62
Andreas Haratsch, Christian König, Matthias Pechstein, Europarecht, recital 470 (12^th^ ed Mohr Siebeck 2020).

63
*Id*.

64 Judgment of the Court (Sixth Chamber) of 3 February 1994, Office National de l’Emploi v Madeleine Minne, C-13/93, ECLI:EU:C:1994:39, recital 17.

65 Calliess/Ruffert/Schmalenbach, 6. Aufl. 2022, AEUV Art. 351 Rn. 21.

66 Judgment of the Court of 30 April 1974, R. & V. Haegeman v Belgian State, C-181/73, ECLI:EU:C:1974:41, recital 2/6.

67 Jessica M. Wyndham, Margaret W. Vitullo, *Define the Human Right to Science*, 362 Science 975 (2018).

68 Buchholtz/Stenzel in Gierschmann/Schlender/Stentzel/Veil, 1. Aufl. 2017, DS-GVO Art. 5 Rn. 24.

69 Buchner/Petri in Kühling/Buchner, 3. Aufl. 2020, DS-GVO Art. 6 Rn. 1, 10 f.; Pötters in Gola/Heckmann, 3. Aufl. 2022, DS-GVO Art. 5 Rn. 6; Schulz in Gola/Heckmann, 3. Aufl. 2022, DS-GVO, Art. 6 Rn. 2.

70 Kingreen in Calliess/Ruffert, 5. Aufl. 2016, EUV/AEUV Art. 8 GRCh, Rn. 1, 9.

71 Alexander Roßnagel, *Kein “Verbotsprinzip” und kein “Verbot mit Erlaubnisvorbehalt” im Datenschutzrecht*, NJW 2019, 1, 3.

72 Jarass, 4. Aufl. 2021, GRCh Art. 8 Rn. 10.

73 Jarass, 4. Aufl. 2021, GRCh Art. 8 Rn. 11.

74 Roßnagel in Simitis/Hornung/Spiecker gen. Döhmann, 1. Aufl. 2019, DSGVO Art. 5 Rn. 31 ff.

75 Wolff in Schantz/Wolff, Das neue Datenschutzrecht, 2017, Rn. 390.

76 Weichert in Kühling/Buchner, 3. Aufl. 2020, DS-GVO Art. 9 Rn. 4; Schulz in Gola/Heckmann, 3. Aufl. 2022, DS-GVO Art. 9 Rn. 6; Kampert in Sydow/Marsch, 3. Aufl. 2022, DS-GVO Art. 9 Rn. 5.

77 Alexander Roßnagel, *Kein “Verbotsprinzip” und kein “Verbot mit Erlaubnisvorbehalt” im Datenschutzrecht*, NJW 2019, 1, 5.

78 Judgment of the Court (Second Chamber) of 19 October 2016, Patrick Breyer v Bundesrepublik Deutschland, C-582/14, EU:C:2016:779, recital 43 et seq.

79 Rothkegel in Taeger/Gabel/Arning, 4. Aufl. 2022, DS-GVO Art. 4 Rn. 31; Philip Laue, *§ 1 Einführung*, in Das neue Datenschutzrecht in der betrieblichen Praxis, Rn. 16 (Philip Laue, Sascha Kremer, 2^nd^ ed 2019 Nomos).

80 Miranda Mourby, Elaine Mackey, Mark Elliot et al., *Are ‘pseudonymised’ data always personal data? Implications of the GDPR for administrative data research in the UK*, 34 Computer Law & Security Review 222, 228 (2018).

81 Alexander Roßnagel, *Datenlöschung und Anonymisierung*, ZD 2021, 188–192 (189).

82 Fruzsina Molnár-Gábor, *Schutz der Rechte und Freiheiten von Personen bei der Datenverarbeitung im Gesundheitsbereich: Der Risikoansatz der EU-Datenschutz-Grundverordnung (DGSVO)*, 66 Bundesgesundheitsbl 143, 152 (2023).

83 Fruzsina Molnár-Gábor, Michael J.S. Beauvais, Alexander Bernier, Maria P.N. Jimenez, Mikel Recuero, Bartha M. Knoppers, *Bridging the European Data Sharing Divide in Genomic Science*, 24 J Med Internet Res. e37236.

84 Tim Wybitul, Lukas Ströbel, Marian Ruess, *Übermittlung personenbezogener Daten in Drittländer*, ZD 2017, 503–509 (504).

85 Judgment of the Court (Grand Chamber) of 6 October 2015, Maximilian Schrems v Data Protection Commissioner, C-362/14, EU:C:2015:650, recital 73.

86 Christopher Kuner, *Reality and Illusion in EU Data Transfer Regulation Post Schrems*, 18 German Law Journal 881, 902 (2017). Schantz in Simitis/Hornung/Spiecker gen. Döhmann, 1. Aufl. 2019, DS-GVO Art. 45 Rn. 6.

87 Judgment of the Court (Grand Chamber), 8 April 2014, Digital Rights Ireland Ltd v Minister for Communications, Marine and Natural Resources and Others and Kärntner Landesregierung and Others, C-293/12 and C-594/12, EU:C:2014:238, recital 45 et seq.; Opinion of Advocate General Mengozzi delivered on 8 September 2016, Opinion pursuant to Article 218(11) TFEU, Case opinion 1/15, EU:C:2016:656, recital 195 et seq.

88 Regulation (EU) 2016/679 of the European Parliament and of the Council of 27 April 2016 on the protection of natural persons with regard to the processing of personal data and on the free movement of such data, and repealing Directive 95/46/EC (General Data Protection Regulation), Art. 45 (1) and Art. 46 (1) GDPR.

89 European Data Protection Board, *Recommendations 01/2020 on measures that supplement transfer tools to ensure compliance with the EU level of protection of personal data*, adopted on 10 November 2020, p. 3.

90 BeckOK DatenschutzR/Lange/Filip, 40. Ed. 1.11.2021, DS-GVO Art. 46 Rn. 12.

91 Committee on Economic, Social and Cultural Rights (CESCR), Concluding Observations: Canada, 22 May 2006, E/C.12/CAN-CO/4–5, 51.

92 GC No. 25, 9.

93 Committee on Economic, Social and Cultural Rights (CESCR), General comment No. 21 (2009), Right of everyone to take part in cultural life (art. 15, para. 1a of the Covenant on Economic, Social and Cultural Rights), 21 December 2009, E/C.12/GC/21, 18–19, online: https://www.refworld.org/docid/4ed35bae2.html.

94 Committee on Economic, Social and Cultural Rights (CESCR), General Comment No. 20 (2009), Non-discrimination in economic, social and cultural rights, 2 July 2009, E/C.12/GC/20, 13, online: https://www.refworld.org/docid/4a60961f2.html.

95 GC No. 25, 23, 31.

96 Judgment of the Court (Grand Chamber) of 21 December 2011, Air Transport Association of America and Others v Secretary of State for Energy and Climate Change, C-366/10, ECLI:EU:C:2011:864, recital 61; Judgment of the Court of 2 August 1993, Criminal proceedings against Jean-Claude Levy, C-158/91, ECLI:EU:C:1993:332, Judgment of the Court of 14 October 1980, Attorney General v Juan C. Burgoa, C-812/79, ECLI:EU:C:1980:231, recital 9.

97 Micah J. Sheller, Brandon Edwards, G. Anthony Reina et al., *Federated learning in medicine: facilitating multi-institutional collaborations without sharing patient data*, 10 Sci. Rep. 1 (2020).

98 David Froelicher, Juan R. Troncoso-Pastoriza, Jean L. Raisaro et al., *Truly privacy-preserving federated analytics for precision medicine with multiparty homomorphic encryption*, 12 Nat Commun. 5910 (2021).

99 Fruzsina Molnár-Gábor, *Schutz der Rechte und Freiheiten von Personen bei der Datenverarbeitung im Gesundheitsbereich: Der Risikoansatz der EU-Datenschutz-Grundverordnung (DGSVO)*, 66 Bundesgesundheitsbl 143, 153 (2023).

100
*Id*.

101
*Id*.

102 Global Alliance for Genomics and Health, *Ethics review recognition policy*, 13 February 2017, https://www.ga4gh.org/wp-content/uploads/GA4GH-Ethics-Review-Recognition-Policy.pdf.

103 Regulation (EU) 2016/679 of the European Parliament and of the Council of 27 April 2016 on the protection of natural persons with regard to the processing of personal data and on the free movement of such data, and repealing Directive 95/46/EC (General Data Protection Regulation), Recitals 124–138 and Chapter VII, Sections 1 & 2.

104 UN General Assembly, International Covenant on Economic, Social and Cultural Rights, United Nations, Treaty Series, vol. 993, p. 3, 16 December 1966, https://www.refworld.org/legal/agreements/unga/1966/en/33423 [accessed 13 February 2024], Art. 16. Cesare P.R. Romano, Andrea Boggio, *The Right to Benefit from Progress in Science and Technology in World Constitutions* (June 3, 2020). An entry in Max Plank Encyclopedia of Comparative Constitutional Law, Loyola Law School, Los Angeles Legal Studies Research Paper No. 2020–17, Available at SSRN: https://ssrn.com/abstract=3618685.

105 UN General Assembly, International Covenant on Economic, Social and Cultural Rights, United Nations, Treaty Series, vol. 993, p. 3, 16 December 1966, https://www.refworld.org/legal/agreements/unga/1966/en/33423 [accessed 13 February 2024], Art. 18.

